# In-Clinic Versus Web-Based Multidisciplinary Exercise-Based Rehabilitation for Treatment of Low Back Pain: Prospective Clinical Trial in an Integrated Practice Unit Model

**DOI:** 10.2196/22548

**Published:** 2021-03-18

**Authors:** Kamshad Raiszadeh, Jonathan Tapicer, Lissa Taitano, Jonathan Wu, Bahar Shahidi

**Affiliations:** 1 Spinezone Medical Fitness San Diego, CA United States; 2 Department of Orthopaedic Surgery University of California San Diego San Diego, CA United States

**Keywords:** low back pain, telehealth, online therapy, physical therapy, integrated practice unit, rehabilitation

## Abstract

**Background:**

The recent onset of the COVID-19 pandemic has highlighted the need to reduce barriers to access physical therapy and associated care through the use of web-based programs and telehealth for those seeking treatment for low back pain (LBP). Despite this need, few studies have compared the effectiveness of clinic-based versus web-based or telehealth services.

**Objective:**

This study aims to compare the clinical outcomes of clinic-based multidisciplinary therapy in an integrated practice unit (C-IPU) model with online integrated multidisciplinary therapy (O-IPU) in individuals undergoing conservative care for LBP.

**Methods:**

A total of 1090 participants were prospectively recruited to participate in a clinical trial registry (NCT04081896) through the SpineZone rehabilitation IPU program. All participants provided informed consent. Participants were allocated to the C-IPU (N=988) or O-IPU (N=102) groups based on their personal preferences. The C-IPU program consisted of a high-intensity machine-based core muscle resistance training program, whereas the O-IPU program consisted of therapist-directed home core strengthening exercises through a web-based platform. Changes in LBP symptom severity (Numeric Pain Rating Scale), disability (Oswestry Disability Index), goal achievement (Patient-Specific Functional Scale), and frequency of opioid use were compared between the C-IPU and O-IPU groups using multivariate linear regression modeling adjusted for age, gender, treatment number, program duration, and baseline pain and disability.

**Results:**

Approximately 93.03% (1014/1090) of the participants completed their recommended programs, with no group differences in dropout rates (*P*=.78). The C-IPU group showed greater pain relief (*P*<.001) and reductions in disability (*P*=.002) than the O-IPU group, whereas the O-IPU group reported greater improvements in goal achievement (*P*<.001). Both programs resulted in reduced opioid use frequency, with 19.0% (188/988) and 21.5% (22/102) of participants reporting cessation of opioid use for C-IPU and O-IPU programs, respectively, leaving only 5.59% (61/1090) of participants reporting opioid use at the end of their treatment.

**Conclusions:**

Both in-clinic and web-based multidisciplinary programs are beneficial in reducing pain, disability, and opioid use and in improving goal achievement. The differences between these self-selected groups shed light on patient characteristics, which require further investigation and could help clinicians optimize these programs.

**Trial Registration:**

ClinicalTrials.gov NCT04081896; https://clinicaltrials.gov/ct2/show/NCT04081896

## Introduction

### Background

Low back pain (LBP) is a leading cause of disability globally, affecting people of all ages [1-3]. Clinic-based physical therapy visits and other physical activity programs have shown value and are currently the standard of care [4-7]. Internet- and web-based therapies have been increasingly used to implement physical rehabilitation and other behavioral programs [8,9]. Importantly, these platforms have the potential for widespread dissemination at a relatively low cost and convenience for users. This has become even more relevant, as health care practitioners and patients are navigating challenges associated with the COVID-19 pandemic. Recent policy changes during the pandemic have reduced the barriers to telehealth access and have promoted the use of telehealth and web-based platforms for primary, specialty care and physical therapy [10].

There is an abundance of commercially available apps offering pain management and exercises (eg, Kaia, Physera, Hinge, Curable, etc) for the treatment of LBP. In parallel, a small number of studies have demonstrated that telehealth and web-based platforms can be used to successfully perform health evaluations in individuals with chronic LBP [11,12]. However, to date, little research exists on the outcomes of internet-based physical activity treatment programs. Of the data that do exist, there is modest evidence for improvement in general health care outcomes based on smartphone app use, and systematic reviews have found weak evidence for the beneficial effects of digital interventions in LBP management [9,13,14]. Similar outcomes have been reported in other populations, such as those with heart failure [15] and knee pain [13,16].

Despite prior literature suggesting some clinical efficacy using telehealth or web-based platforms in individuals with LBP in the United States, there is little information on how these platforms compare to similar in-clinic programs. Most rehabilitation programs are administered by physical therapists in a clinical setting, and care is often not coordinated with the medical team, such as an integrated practice unit (IPU) with the overarching goal of high-value health care [17]. In an IPU, care is provided by practitioners with different specialties centered around the patient’s disease process. Multidisciplinary spine programs have been shown to be more effective than physical therapy alone [18]; however, current studies have limited generalizability because of problems with access to and interpretation of evidence [19] and recruitment methods leading to populations that do not match general practice.

### Objectives

In this study, we compared the outcomes of in-clinic and web-based exercise-based multidisciplinary spinal treatment programs administered through an IPU. We hypothesized that in this model, both web-based and in-clinic treatment would result in equivalent improvement of patient outcomes of LBP-related symptom severity, disability, goal achievement, and frequency of opioid use. A secondary hypothesis was that individuals would self-allocate based on the severity of symptoms at baseline and that more complex or severely debilitated patients would be more likely to select the in-clinic program.

## Methods

### Study Population

**Figure 1:**
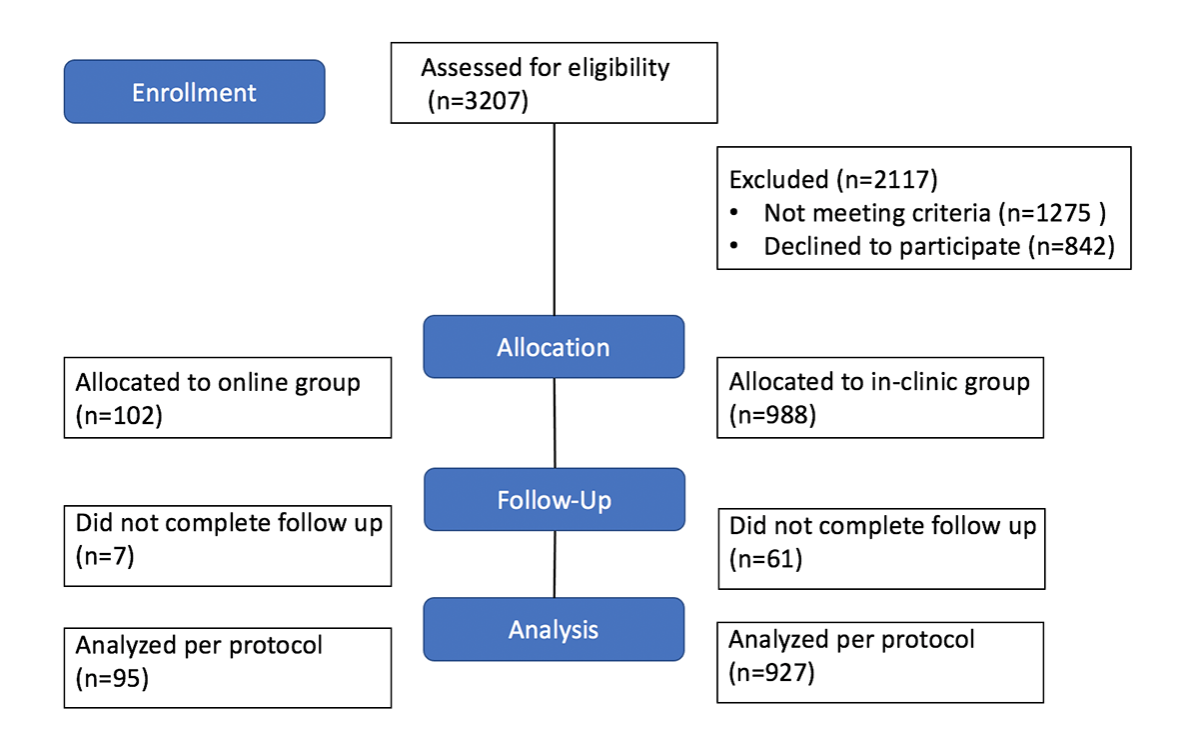
Schematic of participant enrollment.

This was a prospective cohort study using a consecutively enrolled convenience sample of individuals referred to the SpineZone rehabilitation program by their primary care physician. These participants were prospectively recruited to participate in a clinical trial registry (NCT04081896) between January 1 and June 30, 2019. All participants provided informed consent according to the approved institutional review board and the Declaration of Helsinki. Participants were eligible for inclusion if they were aged between 18 and 85 years and were seeking care for symptoms of LBP, including diagnoses of stenosis, disc degeneration, spondylolisthesis, scoliosis, vertebral fracture, radiculopathy, and nonspecific LBP. All participants who completed the initial evaluation questionnaires, including the Oswestry Disability Index (ODI) [20,21], the Numeric Pain Rating Scale (NPRS) [22,23] for pain, and a modified Patient-Specific Functional Scale (PSFS) [24] for goals before initiation of rehabilitation and participated in at least two sessions of either web-based or in-clinic treatment beyond the initial evaluation were included. The ODI is a self-report questionnaire that represents disability as a result of LBP and has been validated in this population [25-27]. The NPRS provides information on the intensity of pain experienced in the back or leg (in the case of radiating symptoms) and has also been validated in patients with LBP [22,23,28]. The PSFS is a self-report questionnaire that identifies patient-prioritized functional activities that are used to establish goals and has been validated in this population [24,29]. Participants self-allocated to either an in-clinic program or a web-based program based on personal preferences. Participants were educated about the time necessary to build muscle and encouraged to participate in as much of the 12-week program as possible. Therefore, to best evaluate the influence of a consistently administered program and to reduce the confounding effects of potential gaps in care on outcomes of interest, participants who had completed their treatment program within 6 months of initiation were included in the analysis (Figure 1). The duration of the program was allowed to vary according to the patient’s needs, and the total number of visits and program duration was documented to account for this variability. For participants who reported symptom resolution before the recommended 12 weeks, the postassessment was conducted at the last attended visit.

### IPU Model

IPUs are a part of the strategy for high-value health care [17], along with measuring outcomes, bundled payments for the full cycle of care, integrated care, and ability for geographic outreach. Our IPU model consisted of a multidisciplinary treatment team, including physical therapists, orthopedic spine surgeons, spine-trained physician assistants, pain specialist consultants, and the clinical director for both in-clinic and web-based practitioners. Multidisciplinary conferences were carried out weekly with the team, and suggestions regarding different areas of focus, modification of exercise program or educational content, further evaluation, need for injections or medication, or need for surgical consultation were discussed and communicated with the primary physician. Physical therapy was administered by therapists trained in psychologically informed treatment strategies [30]. All available radiographic studies were reviewed by a physician assistant and surgeons, and patients were provided information on the natural history of common radiographic findings in the spine, as well as the prevalence of asymptomatic radiologic findings.

Patients in both programs were provided with customized educational materials regarding their condition, sleep, mindfulness, nutrition, posture, and ergonomics via the web-based application. Psychosocial risk factors include catastrophizing, fear avoidance, magnification, depression, and anxiety [30]. At-risk patients underwent education on expressive writing and cognitive behavioral techniques [31]. Group mindfulness classes were also encouraged in patients who were thought to exhibit psychosocial risk factors.

### Clinic-Based IPU Group Protocol

Participants allocated to the in-clinic IPU program (C-IPU) underwent an initial history and standard physical examination by a licensed physical therapist. This examination also included a postural assessment and measurement of isometric lumbar extension strength using a Med-X isokinetic dynamometer [32]. On the basis of these initial assessments, the physical therapist prescribed and progressed machine-based resistance core strengthening exercises, as previously described in detail [33].

### Online IPU Group Protocol

Participants allocated to the online IPU (O-IPU) underwent an initial history and virtual physical examination by a licensed physical therapist or physical therapy aide. On the basis of general goals (activities of daily living improvement or sports and performance), symptom presentation (back pain, back pain with radiating symptoms, and back pain with radiating symptoms that improved with extension), and acuity of symptoms (acute versus chronic), patients were assigned specific directional preference exercises and core strengthening exercises that best matched their symptomatology. Directional preference and home core strengthening exercises were administered on a customized web-based platform along with condition-specific educational content. The exercises assigned on the web-based platform were implemented using a custom mobile-enabled platform that provided images and videos of exercises with written instructions and tracking for a number of sets and repetitions. The number of times logged in and whether the patient accessed the educational material was documented through the platform.

### Outcome Measures and Statistical Analysis

The primary predictor variable of interest was group assignment (web-based vs in-clinic). The primary outcomes of interest included improvements in pain as measured by the change in NPRS score, improvements in LBP-related disability as measured by change in ODI, improvements in goal achievement as measured by change in the PSFS score, and changes in pain medication usage as measured by the frequency of opioid usage according to the following categories: none, <1 per day, 1 to 2 per day, 3 to 5 per day, and >6 per day. Demographic variables such as age, gender, chronicity of symptoms, and history of prior treatment were collected. Chronicity of symptoms was dichotomized into acute (≤3 months duration) and chronic (>3 months duration) symptoms, and prior treatments were categorized as none, conservative care (1, 2, or 3 different modalities), injections, or spinal surgery. Conservative care modalities were defined as physical therapy, acupuncture, or chiropractic treatment. All information was collected by the software platform and confirmed by the physical therapist performing the initial evaluations, re-evaluations, and discharge assessments. For the continuous descriptive variables (demographics and baseline characteristics) and outcomes of interest (change in pain, disability, and goal achievement), univariate and adjusted linear regression models were generated for each outcome, with adjustments including covariates of age, number of visits, total program duration, and baseline levels of pain, disability, or goals. For the categorical or binary descriptive variables (demographics and baseline characteristics) and outcomes of interest (opioid use frequency), chi-square or logistic regression models were used on an as-treated basis. All statistical analyses were performed using SPSS software (version 26.0.0). Statistical significance was set to a *P* value of .05.

## Results

### Participant Characteristics

A total of 1090 participants were included in the analysis. The average age of participants was 62.3 (SD 16.49) years, with 58.81% (641/1090) of participants being female. The mean pain levels were moderate (4.96, SD 1.78 points), as was the average LBP-related disability (26.92, SD 14.26 points) at baseline. Most participants (948/1090, 86.97%) reported symptom durations of greater than 3 months across both groups, indicating chronic symptoms. There were no differences in the proportion of chronic symptoms between the groups (*P*=.47). Most participants reported seeking other treatments before initiating the current program, with 30.46% (332/1090) having received a single modality of prior conservative treatment (physical therapy, chiropractic, or acupuncture), 10.55% (115/1090) having received 2 modalities, and 5.41% (59/1090) having received 3 or more modalities. Some participants reported more invasive prior interventions, such as injections (190/1090, 17.43%) or prior spine surgery (77/1090, 7.06%). Approximately one-third (317/1090, 29.08%) of the participants reported no prior treatment. A greater proportion of participants in the web-based group had received prior treatment (*P*<.001), with more having received 2 (*P*<.001) or ≥3 (*P*=.03) different modalities of conservative treatment. There were no differences in the proportions of participants who had received a single conservative treatment modality (*P*=.85), injections (*P*=.42), or surgery (*P*=.09) between the groups. In addition, the majority (821/1090, 75.32%) of participants were not taking any opioid medications at the time of initiating treatment, with 11.46% (125/1090) reporting taking opioids <1 per day, 7.98% (87/1090) taking 1 to 2 per day, 4.22% (46/1090) taking 3 to 5 per day, and 1.00% (11/1090) taking ≥6 per day. There were no significant differences in opioid use frequency at baseline between groups (*P*=.29). The in-clinic group reported higher levels of pain (*P*=.03) and LBP-related disability (*P*<.001) at baseline than the web-based group (but had higher scores on their goal achievement at baseline (*P*<.001). The participants who enrolled in the web-based program participated in the program for a shorter duration (*P*<.001) but participated in more visits than the in-clinic participants (*P*<.001). More participants in the O-IPU group were diagnosed with lumbar radiculopathy, and more participants in the C-IPU group were diagnosed with degenerative disc disease (*P*<.001). A comparison of the baseline characteristics for each group is shown in Table 1.

**Table 1:** Baseline characteristics between online integrated practice unit and in-clinic integrated practice unit groups.

Variable and group			Value	*t* test or *χ*^2^ (*df*)	*P* value	Difference
**Numeric Pain Rating Scale initial (points), mean (SD)**				−2.25 (1088)	*.03* ^a^	−0.42
	Web-based		4.58 (1.73)			
	In clinic		5.00 (1.78)			
**Oswestry Disability Index initial (points), mean (SD)**				−3.99 (1088)	*<.001* ^a^	−5.87
	Web-based		21.50 (12.09)			
	In clinic		27.47 (14.36)			
**Patient-Specific Functional Scale score (points), mean (SD)**				−3.74 (1088)	*<.001* ^a^	−0.80
	Web-based		3.10 (2.11)			
	In clinic		3.90 (2.04)			
**Visit number (days or log-ins), mean (SD)**				5.92 (1088)	*<.001* ^a^	12.56
	Web-based		36.62 (9.87)			
	In clinic		24.06 (21.18)			
**Program duration (days), mean (SD)**				−13.55 (1088)	*<.001* ^a^	−46.47
	Web-based		45.73 (6.49)			
	In clinic		92.19 (34.55)			
**Age (years), mean (SD)**				1.38 (1088)	.17	2.37
	Web-based		64.41 (11.79)			
	In clinic		62.04 (16.89)			
**Gender (female), n (%)**				2.18 (1)	.14	8.31
	Web-based, n=102		52 (51.20)			
	In clinic, n=988		588 (59.50)			
**Symptom duration >3 months, n (%)**				0.50 (1)	.50	2.50
	Web-based, n=102		91 (89.20)			
	In clinic, n=988		857 (86.70)			
**Opioid use frequency initial, n (%)**						
	**None**			0.001 (1)	.97	−0.19
		Web-based, n=102	77 (75.49)			
		In clinic, n=988	744 (75.30)			
	**<1 per day**			0.05 (1)	.82	0.72
		Web-based, n=102	11 (10.78)			
		In clinic, n=988	114 (11.50)			
	**1 to 2 per day**			0.68 (1)	.41	2.32
		Web-based, n=102	6 (5.88)			
		In clinic, n=988	81 (8.20)			
	**3 to 5 per day**			0.13 (1)	.72	−0.75
		Web-based, n=102	5 (4.90)			
		In clinic, n=988	41 (4.15)			
	**>6 per day**			2.34 (1)	.13	−2.13
		Web-based, n=102	3 (2.94)			
		In clinic, n=988	8 (0.81)			
**Prior treatments, n (%)**						
	**None**			78.82 (1)	*<.001* ^a^	19.10
		Web-based, n=102	12 (11.80)			
		In clinic, n=988	305(30.90)			
	**1 modality**			0.04 (1)	.85	2.30
		Web-based, n=102	29(28.40)			
		In clinic, n=988	303(30.70)			
	**2 modalities**			10.93 (1)	*<.001* ^a^	−10.00
		Web-based, n=102	20(19.60)			
		In clinic, n=988	95(9.60)			
	**3 or more modalities**			4.75 (1)	*.03* ^a^	−4.80
		Web-based, n=102	10(9.80)			
		In clinic, n=988	49(5.00)			
	**Injections**			0.64 (1)	.42	−2.40
		Web-based, n=102	20(19.60)			
		In clinic, n=988	170(17.20)			
	**Surgery**			2.84 (1)	.09	−4.10
		Web-based, n=102	11(10.80)			
		In clinic, n=988	66(6.70)			
**Diagnosis, n (%)**						
	**Lumbar radiculopathy**			40.51 (1)	*<.001* ^a^	−21.00
		Web-based, n=102	32(31.40)			
		In clinic, n=988	103 (10.40)			
	**Nonspecific low back pain**			1.70 (1)	.19	−20.10
		Web-based, n=102	54 (52.90)			
		In clinic, n=988	324 (32.80)			
	**Spondylolisthesis**			3.34 (1)	.07	−5.80
		Web-based, n=102	4 (3.90)			
		In clinic, n=988	96 (9.70)			
	**Stenosis**			3.10 (1)	.08	6.30
		Web-based, n=988	96 (5.90)			
		In clinic, n=988	119 (12.20)			
	**Scoliosis**			2.69 (1)	.10	3.40
		Web-based, n=102	1 (1.00)			
		In clinic, n=988	43 (4.40)			
	**Degenerative disc disease**			25.32 (1)	*<.001* ^a^	23.70
		Web-based, n=102	5 (4.90)			
		In clinic, n=988	283 (28.60)			
	**Fracture**			0.40 (1)	.40	1.90
		Web-based, n=102	0 (0.00)			
		In clinic, n=988	19 (1.90)			

^a^Italics indicate significant difference between online and in clinic groups.

### Clinical Outcomes

**Figure 2:**
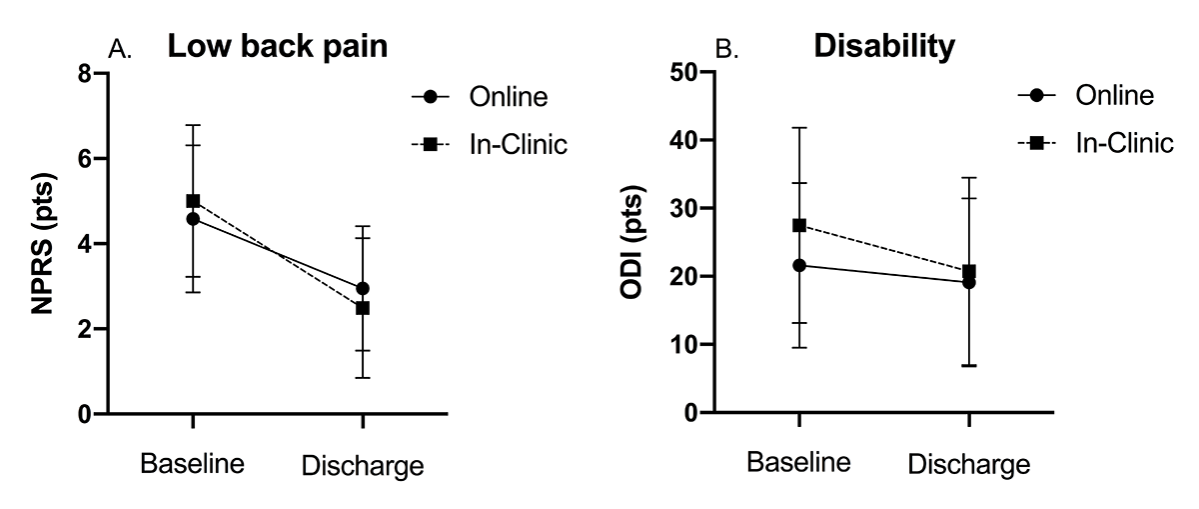
Mean and SDs for low back pain (A) and disability (B) between O-IPU (online) and C-IPU (in-clinic) groups at treatment baseline and discharge. IPU: integrated practice unit; NPRS: numeric pain rating scale; ODI: Oswestry disability index.

**Figure 3:**
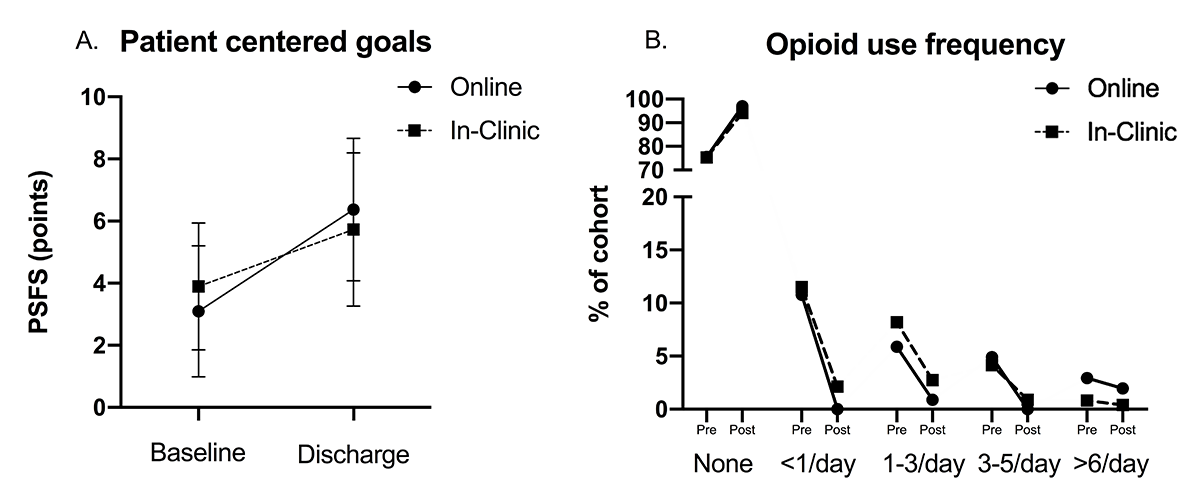
Means and standard deviations for patient opioid use frequency (A) and goal achievement (B) between O-IPU (online) and C-IPU (in-clinic) groups at treatment baseline and discharge. IPU: integrated practice unit; PSFS: patient-specific functional scale.

More than 93% of participants completed their recommended program, with no differences in dropout between the groups (*P*=.78). For the primary outcome of pain improvement, both groups achieved clinically significant reductions, with the in-clinic group demonstrating a significantly greater improvement in pain compared with the web-based group. However, although these differences were statistically significant, they were not clinically significant (mean difference 1.02 points, SE 1.36; *P*<.001; Figure 2). Similarly, the in-clinic group demonstrated statistically larger improvements in LBP-related disability, but these group differences did not reach clinical significance (mean difference 4.26 points, SE 0.32; *P*=.002; Figure 2) .Overall, participants achieved 15.62% (SD 63.6) reductions in LBP-related disability from their baseline scores. Despite greater improvements in pain and disability in the C-IPU group, the O-IPU group reported greater improvements in goal achievement (mean difference 1.70; *P*<.001; Figure 3). The mean change scores and results for the univariate comparisons are reported in Tables 2 and 3. These findings did not change when the models were corrected for age, program visits or duration, or levels of baseline pain or disability, with the exception of goal achievement, where the group differences lost significance but retained a trend (*P*=.06; Table 4). Finally, we observed substantial reductions in the frequency of opioid use for both the in-clinic and web-based programs, with 18.92% (187/988) and 21.56% (22/102) of participants reporting cessation of opioid use on completion of the in-clinic and web-based programs, respectively, leaving only 5.59% (61/1090) of participants reporting opioid use at the end of their treatment (Figure 3). There were no significant differences in the reduction in opioid use frequency between the groups (*P*=.97).

**Table 2:** Unadjusted comparisons for primary outcomes of interest between online integrated practice unit and in-clinic integrated practice unit groups.

Variable and group		Value, mean (SD)	*t* test (*df*)	*P* value	Difference
**Change in pain (points)**			4.64 (1020)	*<.001* ^a^	1.02
	Web-based	−1.97 (1.57)			
	In clinic	−2.99 (2.10)			
**Change in Oswestry Disability Index (points)**			3.15 (1020)	*.002* ^a^	4.26
	Web-based	−2.97 (10.96)			
	In clinic	−7.23 (12.93)			
**Change in Patient-Specific Functional Scale (points**)			5.38 (1020)	*<.001* ^a^	1.70
	Web-based	3.14 (3.13)			
	In clinic	1.44 (2.72)			

^a^Italics indicate significant difference between online and in clinic groups.

**Table 3:** Unadjusted comparisons for change in opioid use frequency between online integrated practice unit and in-clinic integrated practice unit groups.

Variable		None	1 to 3 per day	3 to 5 per day	>6 per day	*χ*^2^*(df*)	*P* value
**Change in opioid use frequency, %**						0.56 (3)	.97
	Web-based	21.51	−4.98	−4.90	−0.98		
	In clinic	18.83	−5.47	−3.25	−0.41		

**Table 4:** Results of the multivariate linear regression for primary outcomes of interest.

Model		*ß* coefficient	SE	*t* test (*df*)	*P* value
**Pain improvement**					
	(Constant)	.49	0.31	1.60 (5)	.11
	In clinic	−1.04	0.19	−5.36 (5)	*<.001* ^a^
	Program duration (days)	.01	0.002	3.32 (5)	*.001* ^a^
	Visit number (days per log-ins)	0	0.002	0.16 (5)	.87
	Age (years)	.01	0.003	2.64 (5)	*.009* ^a^
	Baseline pain (points)	−.67	0.03	−24.00 (5)	*<.001* ^a^
**Oswestry Disability Index improvement**					
	(Constant)	2.74	1.92	1.43 (5)	.15
	In clinic	−3.01	1.31	−2.30 (5)	*.02* ^a^
	Program duration (days)	.03	0.01	2.40 (5)	*.02* ^a^
	Visit number (days/log-ins)	−.03	0.02	−1.48 (5)	.14
	Age (years)	.07	0.02	3.02 (5)	*.003* ^a^
	Baseline Oswestry Disability Index (points)	−.46	0.03	−18.17 (5)	*<.001* ^a^
**Goals improvement**					
	(Constant)	6.40	0.43	14.82 (5)	*<.001* ^a^
	In clinic	−.52	0.28	−1.86 (5)	.06
	Program duration (days)	−.01	0.002	−2.12 (5)	*.03* ^a^
	Visit number (days per log-ins)	.01	0.004	1.54 (5)	.13
	Age (years)	−.01	0.005	−2.50 (5)	*.01* ^a^
	Baseline goals (points)	−.77	0.04	−20.97 (5)	*<.001* ^a^

^a^Italics indicate significant difference between online and in clinic groups.

## Discussion

### Principal Findings

This study demonstrates that both the in-clinic and web-based multidisciplinary programs administered by an IPU resulted in reductions in pain, LBP-related disability, and opioid use in individuals seeking conservative management for LBP. However, contrary to our hypothesis, the in-clinic program demonstrated statistical superiority over the web-based program for pain and disability, although these differences did not reach clinical significance. Similarly, although both groups demonstrated improvements in patient-centered goals, the web-based group reported larger improvements in goal achievement. Importantly, this is the first study to demonstrate reductions in opioid usage in addition to symptom- and function-based outcome measures using a web-based platform.

### Comparisons Between the C-IPU and O-IPU Populations

The results of this study confirm our secondary hypothesis that individuals who self-allocate to the in-clinic program have more severe symptomatology at baseline than those who allocate to the web-based program, with the in-clinic participants demonstrating higher levels of baseline pain and disability. This may explain the larger improvements in pain and disability in the C-IPU group, although the retention of group differences after correcting for baseline pain and disability in the multivariate model suggests that this may not be the only explanation. Similarly, the observation that a greater proportion of participants in the O-IPU group had received multiple different modalities of prior conservative treatment compared with the C-IPU group suggests that this population has an interest in exploring alternative treatments to achieve highly personalized goals. The finding that the O-IPU group reported larger improvements in goal achievement supports the concept that individuals who self-select web-based platforms may have different goals and expectations at baseline than those coming to the clinic. In addition, the underlying bias or belief in treatment success of one treatment modality over the other may influence patient-reported outcomes. Further studies are needed to better explore these patient-selected preferences.

### Comparisons Between Clinical Outcomes or Effectiveness and Prior Studies

Few prior studies have compared the effectiveness of web-based and in-clinic rehabilitation in individuals with LBP, and even fewer studies have incorporated a true multidisciplinary component in their program. One prospective single-arm study investigating program compliance and improvements in LBP and knee pain with a 12-week multidisciplinary digital care program incorporating education and sensor-guided exercise therapy (Hinge) and behavioral support with one-on-one remote health coaching found a significant relationship between app engagement and pain reduction [13]. However, no functional outcomes were obtained, and the population was recruited from employees through email, direct mail, and posters, which may not necessarily represent the general clinical population seeking treatment for LBP.

Of the studies comparing web-based and standard treatments, the results are conflicting. Toelle et al [14] performed a randomized controlled trial comparing an app-based (Kaia) back pain treatment program with a combination of physiotherapy and web-based education and found that the app-based treatment resulted in greater improvements in pain but no group differences in functional ability. However, the treatment frequency in the app-based group was 3 times per week for 12 weeks, whereas the treatment frequency in the physiotherapy group was 1 visit per week for 6 weeks, which may have resulted in an exposure bias in favor of the web-based platform. Indeed, when pain was compared at the 6-week time point (at the end of the physiotherapy group treatment program), both groups demonstrated similar symptoms. Other studies demonstrated no differences across treatment groups; in a randomized controlled trial comparing a web-based app (FitBack) with a wait-listed control group and an alternative care group receiving web-based educational materials via email [34], although the app-based treatment resulted in significantly lower odds of reporting back pain, along with improved functionality, quality of life, and well-being at 4 months posttreatment compared with the control group, there were no differences in these outcomes compared with the alternative care group. Similarly, Mbada et al [35] compared clinic-based McKenzie therapy versus telerehabilitation and found no significant difference in pain, disability, or quality of life between treatment groups. Of note, the McKenzie-based directional preference exercises were also used in this study for patients suspected of having disc pathology irritating or compressing neural structures.

### Patient Population and Treatment Program Methodology

The results of this study demonstrating that the in-clinic program demonstrates statistical superiority for the outcomes of pain and disability are in contrast to other studies reporting the equivalence or superiority of a web-based program. These differences may be because of the patient populations recruited as well as the program design and comparison groups studied. For example, all patients in this study were referred to the program by their primary care physician after the failure of initial treatment with anti-inflammatory medication and education. In prior studies, participants were recruited using methods such as Facebook or other web-based advertisements [14], employer referrals [13], or employer wellness programs [34]. These recruitment methods may not be as generalizable to the standard population of patients with LBP seen by primary care physicians in medical group settings. In addition, one study excluded participants who had received medical care before enrolling in an intervention [34]. Overall, these recruitment methods may have resulted in a selection bias toward a more acute or less severe patient population. Indeed, Toelle et al [14] acknowledged that their study population demonstrated high levels of functional ability in both groups at baseline.

Another difference between this study and prior literature is the program design and methodology. Although many of the app-based platforms incorporate various factors related to back pain within the context of a biopsychosocial disease model, the use of a multidisciplinary approach for exercise-based rehabilitation with continued feedback through active engagement of an integrated care team has not been investigated in prior literature. For example, Bailey et al [13] used a sensor-guided exercise program as well as one-on-one remote health coaching using certified health coaches (through National Board for Health and Wellness Coaching), but patients were not continuously monitored by a multidisciplinary team over the course of their treatment. Similarly, Irvine et al [34] and Toelle et al [14] used predominantly app-based treatment and physical therapists for the control group but did not incorporate routine monitoring by other care providers as part of the treatment progress. In this study, both groups underwent physical therapy administered by psychologically informed practitioners [30], and patients who did not progress were reviewed in a weekly multidisciplinary conference with the physician assistants and surgeons to make adjustments to care, including the need for diagnostic studies, injections, or surgical intervention.

### Study Limitations

This study had several limitations. First, it did not employ a true no-treatment control group, making the natural history effects of the treatment difficult to rule out. However, given that the goal of this study was to determine whether web-based implementation would provide similar benefits to in-clinic rehabilitation, the lack of a control group should not influence the primary study hypothesis. Second, this study employed a pragmatic study design, in which participants were not randomly allocated to treatment groups, introducing the possibility of selection bias. Indeed, some differences in baseline characteristics (eg, pain, disability) were observed between the groups. However, our statistical approach of adjusting for these baseline differences allowed us to correct for some of these discrepancies. Second, it also allows us to gain a better understanding of the factors influencing patient preferences in choosing care. Finally, although both groups experienced reduced pain, opioid use, and improved goal achievement that reached clinical relevance (determined by minimal clinically important difference values) [20-25,28], the reductions in disability did not reach clinical significance. However, given the concurrent reductions in opioid use in a proportion of patients, the overall reductions in pain and disability may be underestimated because of decreases in pharmacological management.

### Conclusions

This study found that C-IPU and O-IPU programs administered by a multidisciplinary team in an IPU both resulted in reductions in symptom severity, LBP-related disability, and opioid use frequency as well as improvements in goal achievement. The C-IPU was statistically superior to the O-IPU group in reducing pain and disability, and the O-IPU group was statistically superior in improving patient-specific goal achievement. Both programs resulted in equivalent and substantial reduction in opioid use frequency, which is a priority area in a population that is at high risk for developing opioid dependence.

## Abbreviations

C-IPU: clinic integrated practice unit
IPU: integrated practice unit
LBP: low back pain
NPRS: Numeric Pain Rating Scale
ODI: Oswestry Disability Index
O-IPU: online integrated practice unit
PSFS: Patient-Specific Functional Scale
